# Lurasidone-induced mania: A case report

**DOI:** 10.1192/j.eurpsy.2022.1024

**Published:** 2022-09-01

**Authors:** T. Fernández, L. Navarro, O. Marco, L. Tardon, N. Arbelo, L. Ilzarbe, P. Barrio, I. Pacchiarotti

**Affiliations:** Hospital Clínic Barcelona, Psychiatry, Barcelona, Spain

**Keywords:** mania, switch, lurasidone

## Abstract

**Introduction:**

Lurasidone is an atypical antipsychotic agent with potential antidepressant effects through its antagonist activity at the 5-HT7 receptor. Although treatment-emergent affective switch (TEAS) induced by second-generation antipsychotics are not frequent, several cases have been reported. To our knowledge, there is no evidence of lurasidone-induced TEAS.

**Objectives:**

To describe a case of lurasidone-induced mania.

**Methods:**

We describe a clinical case of a patient admitted to our psychiatric outpatient unit who developed a manic episode, presumably induced by the introduction of lurasidone. We also conduct a review of the literature on this subject.

**Results:**

A 37-year-old man diagnosed with obsessive-compulsive disorder (OCD) and an alcohol use disorder was hospitalized due to OCD decompensation with depressive symptomatology and suicidal thoughts, and for alcohol detoxification process. Since he had a previous history of clomipramine-induced TEAS, he was started on lurasidone up to 111mg to avoid the use of antidepressants, showing a progressive improvement of depressive symptoms. Thus, the patient was discharged when alcohol detoxification process was completed. Eight days after discharge, the patient began to show manic symptoms, so he had to be readmitted. Lurasidone was discontinued and valproic acid up to 1000mg/day as mood stabilizer was added, presenting a positive remission of manic symptoms.

**Conclusions:**

According to our experience, lurasidone may have induced an affective switch in this patient. Based on our findings, patients and psychiatrists should monitor possible lurasidone-induced mood switching. However, further research is needed in order to back-up this one case report findings.

**Disclosure:**

No significant relationships.

